# Heat shock protein 90 chaperone activity is required for hepatitis A virus replication

**DOI:** 10.1128/jvi.00502-25

**Published:** 2025-06-05

**Authors:** You Li, Xin Zheng, Ling Xie, Maryna Kapustina, Takayoshi Shirasaki, Bryan Yonish, Xian Chen, Asuka Hirai-Yuki, Noriyo Nagata, Ryosuke Suzuki, Masanori Isogawa, Matthew R. Vogt, Masamichi Muramatsu, Stanley M. Lemon

**Affiliations:** 1 Department of Pediatrics, The University of North Carolina at Chapel Hill 2331, Chapel Hill, North Carolina, USA; 2 Department of Virology II, National Institute of Infectious Diseases, Japan Institute for Health Security, Tokyo, Japan; 3 Department of Infectious Disease Research, Foundation for Biomedical Research and Innovation at Kobe, Hyogo, Japan; 4 Research Center for Biosafety, Laboratory Animal and Pathogen Bank, National Institute of Infectious Diseases, Japan Institute for Health Security, Tokyo, Japan; 5 Department of Biochemistry and Biophysics, The University of North Carolina at Chapel Hill 2331, Chapel Hill, North Carolina, USA; 6 Department of Cell Biology and Physiology, The University of North Carolina at Chapel Hill 2331, Chapel Hill, North Carolina, USA; 7 Department of Microbiology and Immunology, The University of North Carolina at Chapel Hill 2331, Chapel Hill, North Carolina, USA; 8 Lineberger Comprehensive Cancer Center, The University of North Carolina at Chapel Hill 2331, Chapel Hill, North Carolina, USA; 9 Department of Pathology, National Institute of Infectious Diseases, Japan Institute for Health Security, Tokyo, Japan; 10 Department of Medicine, The University of North Carolina at Chapel Hill 2331, Chapel Hill, North Carolina, USA; Wake Forest University School of Medicine, Winston-Salem, North Carolina, USA

**Keywords:** HSP90 inhibitor, HSP70, picornavirus, hepatovirus, chaperone, replicon, antiviral, mouse model, quasi-enveloped virus, HD-PTP, ACC1

## Abstract

HSP90 heat shock chaperones are essential for maintaining cellular proteostasis, as well as the ATP-dependent folding and functional maturation of many viral proteins. As a result, inhibitors of HSP90 have broad antiviral activity, disrupting replication of many viruses at concentrations below those causing cytotoxicity. Among the *Picornaviridae*, HSP90 inhibitors block replication of multiple *Enterovirus*, *Aphthovirus,* and *Cardiovirus* species, in some cases, by preventing post-translational processing and assembly of P1 capsid proteins. Hepatitis A virus (HAV), classified within the genus *Hepatovirus*, has been suggested to be an exception among picornaviruses and to replicate independently of HSP90, possibly because its slow translational kinetics could facilitate co-translational folding and assembly of its capsid proteins. However, we show here that HAV replication is highly dependent upon HSP90, both in human hepatocyte-derived cell lines, in which the 50% inhibitory concentration of geldanamycin was 8.7–11.8 nM, and *in vivo* in *Ifnar1^-/-^* mice. Label-free proteomics experiments suggested that HSP90 interacts with capsid proteins or their precursors and may thus facilitate the folding and assembly of capsid proteins, as it does for enteroviruses and aphthoviruses. By contrast, there was no evidence for HSP90 interacting with any nonstructural protein, and HSP90 inhibitors did not impair 3C^pro^ proteolytic activity. Despite this, and in contrast to previous studies of enteroviruses and aphthoviruses, geldanamycin potently inhibited replication of a subgenomic HAV replicon. We conclude that HAV is no exception from the HSP90-dependent nature of other picornaviruses and indeed is more dependent on HSP90 than other picornaviruses for amplification of its genome.

**IMPORTANCE:**

Hepatitis A virus (HAV), a common cause of acute infectious hepatitis, has been reported to differ from other picornaviruses in not requiring heat shock protein HSP90 for efficient replication. However, we show here that productive HAV infection is highly dependent on HSP90 and that HAV replication is potently blocked both in cell culture and *in vivo* in the murine liver by chemical inhibitors of HSP90. Such inhibitors also disrupt the replication of a subgenomic HAV RNA replicon, indicating that HSP90 is required for the assembly of functional replication organelles. This highlights a key difference from other picornaviruses for which HSP90 is required primarily, if not exclusively, for the maturation of the P1 capsid proteins.

## INTRODUCTION

The heat shock chaperone system is critical for maintaining healthy cellular proteostasis (1, 2). Working in concert with heat shock protein 70 (HSP70) and numerous other co-chaperones, HSP90 proteins are central players in an evolutionarily conserved chaperone network that promotes the post-translational acquisition of function by hundreds of “client” proteins. The HSP90 machinery facilitates the ATPase-dependent folding of client proteins, promotes the assembly of client proteins into multi-protein complexes, and enables the interactions of client proteins with specific ligands (1, 2). Functioning in an integrated fashion with ubiquitin ligases and proteasome-mediated decay, the heat shock network is crucial for the health of eukaryotic cells, not only at times of cellular stress, but also under normal physiological conditions (1, 2).

Both DNA and RNA viruses are universally dependent upon cellular synthetic and metabolic pathways to support the replication of their genomes and the production of new viral particles. Given the vital role of the heat shock chaperone system in maintaining cellular proteostasis, it is not surprising that heat shock proteins also play crucial roles in the folding, assembly, and acquisition of function by viral proteins (3, 4). Numerous studies suggest that many, if not all, viruses are dependent on some component of the heat shock chaperone system. Known protein clients of HSP70 and HSP90 include both structural and nonstructural viral proteins. Examples exist for cellular chaperones supporting virtually every step in the replicative cycle of a virus, including entry, trafficking, virion disassembly, protein synthesis, replication organelle formation, DNA or RNA transcription, and capsid folding and assembly (3, 4). As a consequence, small molecule inhibitors of HSP90, such as geldanamycin and its derivatives, are among the most broadly acting antiviral compounds known, often suppressing viral replication at concentrations significantly below those inducing cytotoxicity (5–9). Such results suggest that viral proteostasis may be more dependent on heat shock chaperones than cellular proteostasis, an attribute that could be linked to exceptionally large quantities of aggregation-prone viral proteins produced in infected cells, the complex and metastable nature of viral capsid assemblies that must be stable, yet capable of disassembly under the right conditions, and the quasispecies nature and accompanying diversity of protein sequences encoded by some viruses (4, 10).

Among the family *Picornaviridae*, experiments with geldanamycin have shown that four different species of enteroviruses, *Enterovirus A* (enterovirus A71, EV-A71), *Enterovirus B* (coxsackievirus B3), *Enterovirus C* (poliovirus type 1), and *Rhinovirus B* (rhinovirus B14, RV-B14), as well as aphthoviruses (foot-and-mouth disease virus, FMDV) and cardioviruses (Theiler’s murine encephalomyelitis virus) are all dependent upon HSP90 for efficient replication (5, 11–14). HSP90 binds to the P1 capsid protein precursor of poliovirus in association with its p23 co-chaperone, promoting the folding required for the P1 precursor to become a competent substrate for the viral 3C^pro^ protease (5). In the absence of HSP90 activity, the P1 precursor misfolds and is targeted for destruction by the proteasome. Thus, geldanamycin has potent anti-poliovirus activity in cell culture and also in poliovirus receptor transgenic mice in which it effectively blocks viral replication and reduces viral load in the central nervous system (5). More recent inhibitor experiments indicate that HSP90 activity is also required for the processing of the aphthovirus P1 capsid precursor (12). In both cases, the replication of subgenomic poliovirus or aphthovirus RNA replicons was not blocked by HSP90 inhibition, suggesting that HSP90 activity is not required for the synthesis or processing of nonstructural proteins from the P2P3 segments of the viral polyprotein or the assembly of productive replication organelles (5, 12).

Hepatitis A virus (HAV), a common cause of acute, enterically transmitted viral hepatitis, represents an additional genus within the *Picornaviridae* (15). Although the role of HSP90 in hepatovirus replication has not been studied in detail, a previous report suggests that geldanamycin does not inhibit productive HAV infection (16). If true, this would make hepatoviruses distinct from other picornaviruses in not requiring HSP90 for the maturation and processing of its capsid proteins. It has been suggested that this difference could be related to slow translation of HAV RNA associated with the extreme codon bias evident in its genome, as this could allow more time for co-translational folding of its capsid proteins and thus less need for heat shock chaperones (10, 16). Here, we show to the contrary that productive HAV infection is highly dependent upon HSP90 activity both in cell culture and *in vivo* in HAV-permissive mice, and that HSP90 interacts specifically with capsid proteins in HAV-infected cells. We also show that replication of a subgenomic HAV RNA replicon requires HSP90, in sharp contrast to the HSP90-independent replication of other picornaviral subgenomic replicons.

## RESULTS

### HAV replication in hepatoma cells requires HSP90 chaperone activity

**Fig 1:**
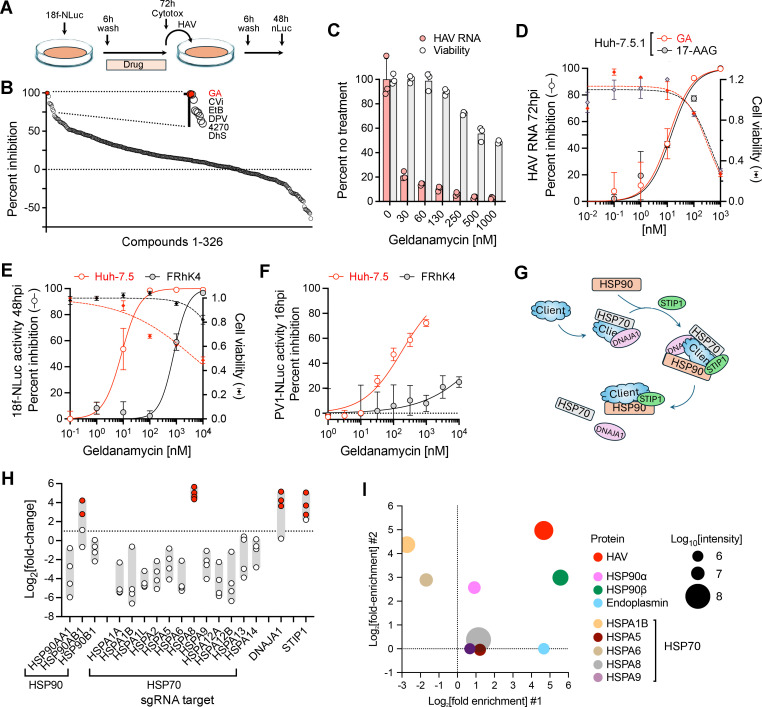
The heat shock pathway is required for HAV replication. (A) Screen for inhibitors of infectious virus production using the 18f-NLuc reporter virus and a commercial 326 chemical compound library. Huh-7.5.1 cells were treated with compounds (10 µM) from 6 to 72 hours after infection, with cell viability assessed at 72 hours. Supernatant fluids collected at 72 hours were assayed for virus by measuring nanoluciferase expressed in a second round infection. Results were normalized for cytotoxicity. (B) Results of the chemical screen. The top six inhibitory compounds are highlighted: GA, geldanamycin; CVi, crystal violet; EtB, ethidium bromide; DPV, dapivirine; 4270, 4270-0405; DhS, dehydroandrographolide succinate. See also Table S1. (C) Geldanamycin inhibition of infectious virus release. Huh-7.5.1 cells were treated, and cell viability was assessed as in panel A. HAV was assayed in supernatant fluids by quantifying viral RNA 48 hours after infection of naive cells. (D) Antiviral activity of HSP90 inhibitors against HM175/18f virus. Huh7.5.1 cells were infected at a multiplicity of 100 GE/cell and treated with increasing concentrations of inhibitors from 6 to 72 hours post-infection. HAV RNA was quantified at 72 hours by RT-qPCR. Dashed lines indicate cell viability with the DMSO-treated control set at 1.0. Geldanamycin (open red symbols) IC_50_ = 11.8 nM, CC_50_ 333 nM; 17-AAG (shaded black symbols) IC_50_ = 14.2 nM, CC_50_ 418 nM. Data are means ± SD, *n* = 3. (E) Antiviral activity of geldanamycin against the 18f-NLuc reporter virus in Huh-7.5 (open red symbols, IC_50_ = 8.7 nM) or FRhK4 (shaded black symbols, IC_50_ = 804 nM) cells. NLuc activity was measured 48 hours post-infection. Dashed lines indicate cell viability with the DMSO-treated control set at 1.0. Data are means ± SD, *n* = 3. (F) Geldanamycin inhibition of NLuc activity expressed by a PV1 reporter virus 16 hours after infection of Huh-7.5 and FRhK4 cells. Huh-7.5 IC_50_ = 183 nM; FRhK4 IC_50_ = >10 µM. (G) Client protein flow through the heat shock pathway. DNAJA1 is a co-chaperone for HSP70. STIP1 facilitates the transfer of client proteins from HSP70 to HSP90 and interacts with both heat shock proteins. (H) Fold increase in individual sgRNAs targeting heat shock proteins in Huh-7.5 cells following selection against lethal HAV infection in a previously described genome-wide CRISPR screen (18). Dashed line indicates a twofold change in sgRNA counts; red symbols, false discovery rate < 0.0005 by ANOVA. (I) Bubble plot showing heat shock proteins identified by mass spectrometry in two independent stable isotope labeling by amino acids (SILAC) analyses of gradient-purified quasi-enveloped eHAV (19). Mean fold enrichment of mass-tagged heat shock proteins identified in virus samples from cells grown in each experiment using media with “heavy” versus “light” isotopes from the first and second SILAC experiments (#1 and #2) are plotted on *X* and *Y* axes, respectively, while the size of bubbles represents the relative mean intensity of peptides.

Two distinct lines of evidence support a requirement for the HSP70/HSP90 chaperone network in HAV replication. First, a chemical screen for inhibitors capable of blocking the replication and release of a nanoluciferase-expressing reporter virus, 18f-NLuc (17), identified geldanamycin, an inhibitor of the heat shock protein HSP90, as the most active of 326 small molecules in a commercial antiviral compound library (TargetMol, Wellesley Hills, USA) (Fig. 1A and B; Table S1). In confirmatory experiments, geldanamycin inhibited greater than 50% of the infectious virus released from Huh-7.5.1 cells at a concentration of 30 nM (Fig. 1C). In assays using RT-qPCR to monitor viral RNA replication, the 50% inhibitory concentration (IC_50_) of geldanamycin was 11.8 nM, 28-fold below the 50% cytotoxic concentration (CC_50_), which was 333 nM (Fig. 1D). Similarly, the IC_50_ of 17-AAG, a chemically related compound that, like geldanamycin, blocks ATP binding to the N-terminal domain of HSP90, was 14.2 nM, far below the CC_50_, which was 418 nM. In similar assays, we assessed the capacity of geldanamycin and 17-AAG to inhibit HAV replication in a T-antigen transformed, adult human hepatocyte cell line, PH5CH8, in which innate immune responses were blocked by genetic knockout of mitochondrial antiviral signaling protein (MAVS) and interferon regulatory factor 1 (IRF1) (17). In these cells, the geldanamycin IC_50_ was 4.3 nM and 17-AAG IC_50_ was 3.2 nM (Table 1).

**TABLE 1:** Antiviral activities of heat shock protein inhibitors

Inhibitor	Mechanism of action		Assay method	IC_50_ (nM)	IC_50_ 95% CI (nM)	CC_50_ (nM)
Huh-7.5 or Huh-7.5.1 cells						
Geldanamycin	Blocks ATP binding to HSP90		RT-qPCR NLuc^*a*^	11.8 8.7	5.0–27.6 6.3–11.2	333.0 > 1,000
17-AAG	Blocks ATP binding to HSP90		RT-qPCR	14.2	6.6–31.8	418.0
HSP990	Inhibits HSP90 ATPase activity		RT-qPCR	2.2	1.6–3.2	56.9
STA9090	Binds HSP90 N-terminal ATP-binding site		RT-qPCR	12.1	3.0–60.2	146.8
TAS116	Binds HSP90 N-terminal ATP-binding site		RT-qPCR	84.3	34.3–216.0	835.2
JG98	Allosteric inhibitor of HSP70		NLuc	534	213–1,435	>1,000
VER155008	Binds HSP70 nucleotide-binding domain		NLuc	3,323	1,269–9,071	>10,000
116-9e	DNAJA-1 inhibitor		NLuc	8,758	3,300–>10,000	>10,000
PH5CH8 cells						
Geldanamycin	Blocks ATP binding to HSP90		RT-qPCR	4.3	3.5–5.3	nd^*b*^
17-AAG	Blocks ATP binding to HSP90		RT-qPCR	3.2	1.8–4.7	nd
293T cells						
Geldanamycin	Blocks ATP binding to HSP90		NLuc	4.7	2.0–10.0	>1,000
17-AAG	Blocks ATP binding to HSP90		NLuc	11.7	5.3–24.6	>1,000
FRhK4 cells						
Geldanamycin	Blocks ATP binding to HSP90		NLuc	804	639–964	>10,000

^*a*^ HAV replication monitored by RT-qPCR quantitation of viral RNA or nanoluciferase (NLuc) expressed by the 18f-NLuc reporter virus.

^*b*^ nd, not done.

These results contrast sharply with the IC_50_ of 5.37 µM reported for geldanamycin inhibition of HAV replication in FRhK4 fetal rhesus kidney cells by Aragonès et al. (16). We thus assessed the capacity of geldanamycin to inhibit replication of the 18f-NLuc reporter virus in FRhK4 cells. These experiments confirmed that HAV replication is relatively resistant to geldanamycin in FRhK4 cells, with an IC_50_ of 804 nM, almost 100-fold higher than the geldanamycin IC_50_ of 8.7 nM in Huh-7.5 cells infected with the same virus under similar conditions (Fig. 1E). Cellular ATP assays suggested that geldanamycin was also less cytotoxic in FRhK4 cells than Huh-7.5 cells in these experiments (CC_50_ 59 µM versus 5.1 µM) (Fig. 1D and E). These results reveal a surprising cell-type-specific difference in the activity of geldanamycin against HAV. To further assess this difference and rule out an HAV-specific effect, we compared the capacity of geldanamycin to suppress replication of a type 1 poliovirus reporter virus, PV1-NLuc, in Huh-7.5 and FRhK4 cells. Although geldanamycin was substantially less active against PV1-NLuc than 18f-NLuc in both cell types, it was approximately 100-fold less active against PV1-NLuc in FRhK4 cells (IC_50_ = >10 µM) than Huh-7.5 cells (IC_50_ = 183 nM, 95% CI 136–250 nM) (Fig. 1F). These results suggest either a lack of drug uptake or a functionally different complement of heat shock chaperones in FRhK4 cells (see Discussion).

A second line of evidence suggesting a requirement for the heat shock chaperone system in HAV replication comes from a previously reported genome-wide CRISPR screen that identified heat shock protein HSP70 (HSPA8) as one of 39 candidate high-confidence HAV host factors (18). The heat shock chaperone pathway involves initial binding of a client protein to HSP70, followed by its transfer to HSP90 in a process facilitated by DnaJ homolog subfamily A member 1 (DNAJA1), an HSP70 co-chaperone, and stress-induced phosphoprotein 1 (STIP1) (Fig. 1G). Remarkably, both DNAJA1 and STIP1 were identified in addition to HSPA8 in the CRISPR screen, each with a calculated false discovery rate (FDR) less than 10^−5^ (18).

The CRISPR screen involved a selection process in which guide RNAs (sgRNAs) targeting essential host factors were selected for their ability to prevent HAV-induced cell death (18). The Brunello lentivirus sgRNA library used in the screen included four distinct sgRNAs for each of 19,114 genes. In light of the chemical inhibitor data described above, we reviewed the extent to which individual sgRNAs targeting HSP90 were selected. Huh-7.5 cells express three major HSP90 proteins with ATPase activities: HSP90α (encoded by HSP90AA1), HSP90β (HSP90AB1), and endoplasmin (HSP90B1). Three of four sgRNAs targeting HSP90AB1 were positively selected in the CRISPR screen, two at a very high level (*Q* < 10^−5^) (Fig. 1H). By contrast, no sgRNAs targeting HSP90AA1 or HSP90B1 were positively selected. We also reviewed data from a previously published proteomics comparison of gradient-purified quasi-enveloped eHAV versus non-viral extracellular vesicles (EVs) that used stable isotope labeling by amino acids (19). All three major HSP90 proteins were detected in both eHAV and EV samples in two independent experiments, but HSP90β (detected in four out of four samples tested) was consistently more enriched in eHAV versus EVs than HSP90α (detected in only two of four samples) (Fig. 1I). Collectively, these results suggest that productive HAV replication is more dependent on HSP90β than HSP90α. This conclusion is consistent with previous studies showing that HSP90β, not HSP90α, associates with EV-A71 capsid proteins and facilitates virus production (13, 14). Multiple HSP70 proteins were also present in the proteomics study (Fig. 1I). Of these, HSPA8 was the most abundant but equally distributed among eHAV and EV samples.

A targeted screen of additional heat shock protein inhibitors revealed that other inhibitors targeting the amino-terminal ATP-binding site of HSP90 (HSP990, STA9090, and TAS116) are highly active in blocking HAV replication (Table 1). By contrast, chemical inhibitors of HSP70 (JG98 and VER155008) or DNAJA1 (116-9e) demonstrated anti-HAV activity only at concentrations close to their CC_50_.

### Antiviral activity of the HSP90 inhibitor 17-AAG *in vivo*

**Fig 2:**
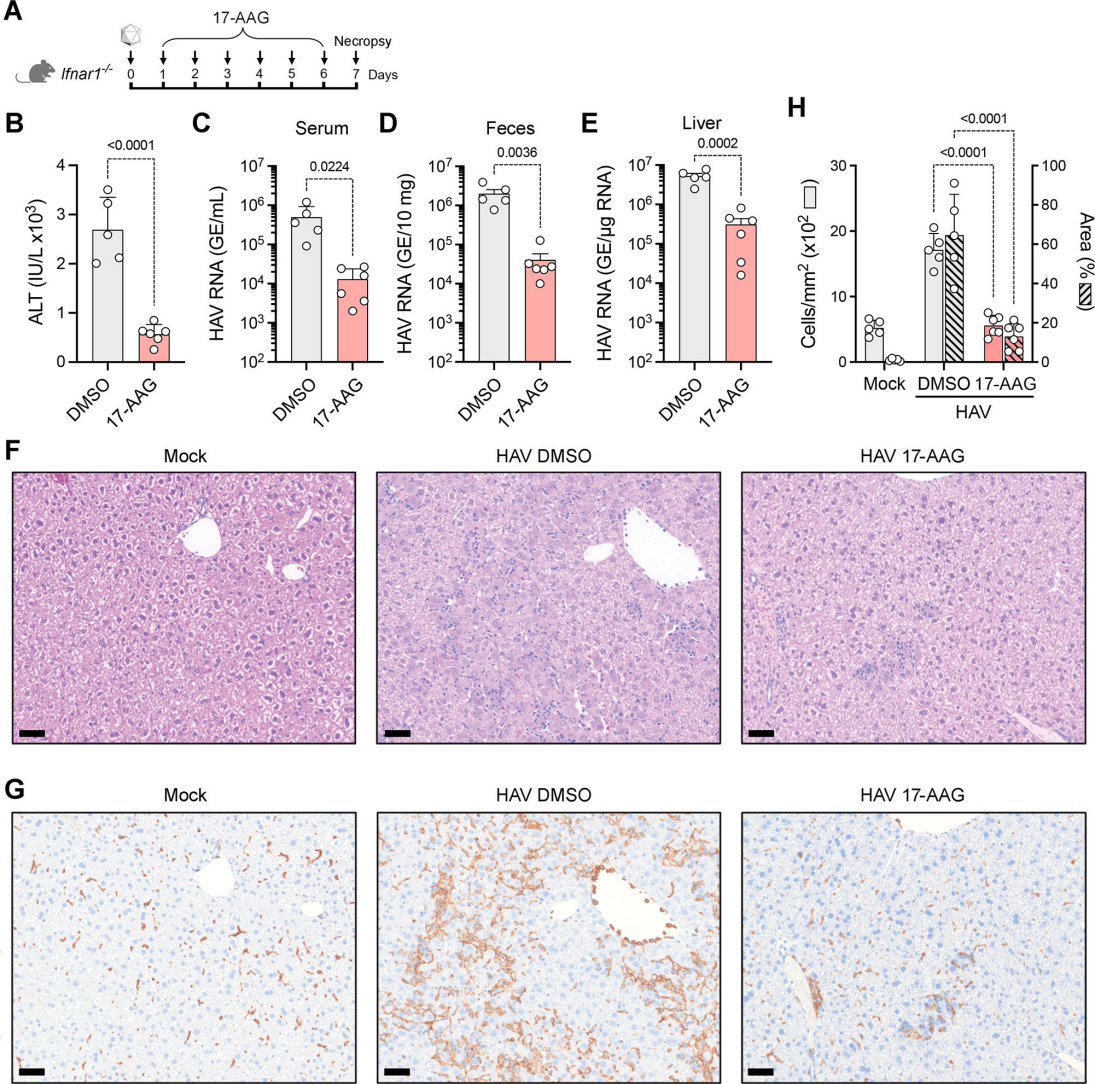
Antiviral activity of the HSP90 inhibitor 17-AAG in infected *Ifnar1^-/-^* mice. (A) Infection scheme: mice infected by i.v. inoculation of 10^6^ GE mouse-passaged HAV were treated with 1 mg 17-AAG or vehicle (DMSO), only administered by i.p. injection daily for 6 days. The experiment was terminated, and mice were necropsied 7 days post-infection (dpi). (B) Serum alanine aminotransferase activities at 7 dpi. (C–E) HAV RNA quantified by RTqPCR in (C) serum, (D) feces, and (E) liver at 7 dpi. *n* = 5–6; *P*-values were calculated by two-way *t*-test. (F) Hematoxylin and eosin-stained sections of liver from (left to right) an uninfected mouse (mock) and infected *Ifnar1^-/-^* mice treated with 17-AAG or vehicle only at 7 dpi. (G) Immunohistochemical staining for macrophages with antibody to ionized calcium-binding adapter molecule 1 in serial sections of mouse livers. (H) Digital image scoring of IBA1-positive cells (cells/mm^2^) and areas of inflammation (percent area) in liver sections from untreated and 17-AAG-treated HAV-infected *Ifnar1^-/-^* mice. Three regions of interest were scanned in each of three different sections from each mouse liver. Liver sections from uninfected mice are included for comparison. *n* = 5–6; *P*-values were calculated by two-way ANOVA. Scale bars = 50 µm.

To ascertain whether HSP90 activity is required for efficient HAV replication *in vivo*, we utilized a small animal model of hepatitis A, administering 17-AAG to *Ifnar1^-/-^* mice 1 day after infection with murine-passaged HM175 virus (Fig. 2A). *Ifnar1^-/-^* mice do not express a functional receptor for type I interferon and are highly permissive for HAV (20). When necropsied 7 days post-infection (dpi), mice given 17-AAG by intraperitoneal injection had significantly lower increases in serum alanine aminotransferase (ALT) activity, a key marker of hepatic inflammation, than control mice receiving vehicle (DMSO) only (Fig. 2B). Similarly, the quantity of viral RNA in serum, feces, and liver was significantly reduced in the 17-AAG treated mice (Fig. 2C through E). Consistent with the reduction in serum ALT activity, inflammatory cell infiltrates were notably fewer and smaller in size in hematoxylin and eosin-stained sections of liver from 17-AAG treated animals (Fig. 2F). We quantified these infiltrates by immunohistochemical staining of liver sections for calcium-binding adapter molecule 1 (IBA1), a macrophage marker protein (Fig. 2G), followed by digital image analysis. Both the number of IBA1-positive cells and the area occupied by IBA-positive cells within the liver section were significantly reduced in 17-AAG-treated animals (Fig. 2G and H). We conclude that HSP90 chaperone activity is required for efficient HAV replication and contributes to the pathogenicity of the virus *in vivo*.

### Steps in the replicative cycle of HAV requiring HSP90 activity

**Fig 3:**
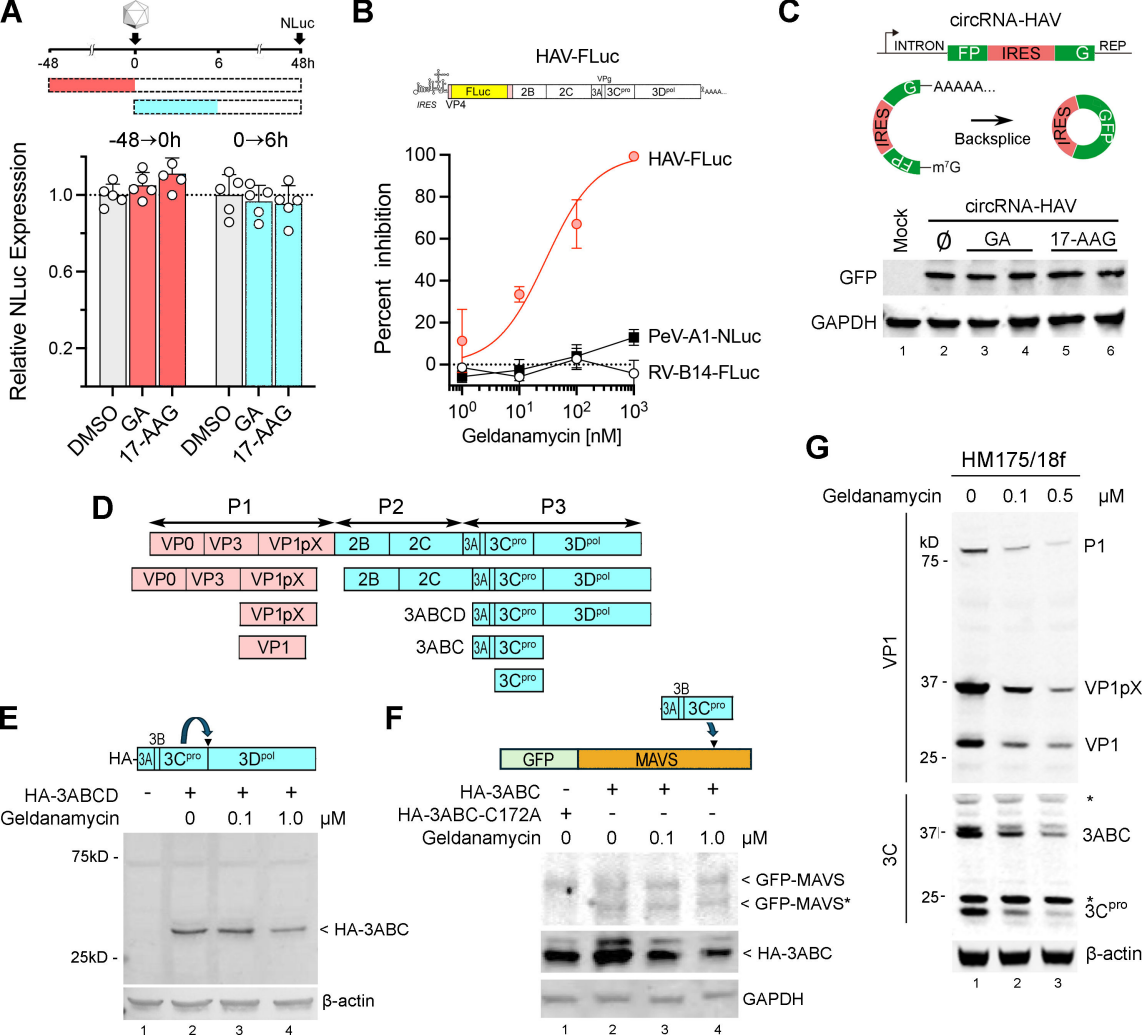
HSP90 activity is required for HAV replication. (A) NLuc expressed by cells treated with the HSP90 inhibitors geldanamycin (GA, 50 nM) or 17-AAG (50 nM) for 48 hours prior to or 6 hours after infection with the 18f-NLuc reporter virus. (B) Inhibition of luciferase expressed by cells transfected with a subgenomic HAV replicon RNA, HAV-FLuc, or similarly constructed human rhinovirus (RV-B14-FLuc) and human parechovirus A1 (PeV-A1-NLuc) replicons, in the presence of increasing geldanamycin concentrations. HAV-FLuc IC_50_ = 29.1 nM (95% CI 12.9–66.1 nM). (C) Immunoblot of GFP expressed by cells transfected with a circular RNA HAV IRES reporter, circRNA-HAV (22), in the presence of geldanamycin (100 nM), 17-AAG (100 nM), or no inhibitor (∅). (D) Polyprotein processing leading to mature VP1 and 3C^pro^ proteins. Primary polyprotein cleavage occurs at the P1/P2 junction between the structural (pink) and nonstructural (blue) protein precursors. All cleavages are catalyzed by 3C^pro^. (E) HA immunoblot of lysates from geldanamycin-treated cells transfected with a vector expressing HA-3ABCD. Only one protein band, HA-3ABC, is apparent. (F) Immunoblots of lysates from cells transfected with vectors expressing GFP-MAVS and HA-3ABC or HAV-3ABC-C172 (catalytically inactive 3C mutant). GFP-MAVS*, 3C^pro^ GFP-MAVS cleavage product. (G) Anti-VP1 and anti-3C immunoblots of proteins present in lysates of cells infected for 72 hours with 18f virus, then treated with the indicated concentration of geldanamycin for 24 hours. *Nonspecific protein band.

We next focused on determining the step in the replicative cycle of HAV at which HSP90 is needed. To exclude a requirement for HSP90 in the attachment or entry of virus into cells, we carried out a time-of-addition experiment, treating cells with geldanamycin or 17-AAG prior to or for 6 hours after infecting the cells with the 18f-NLuc reporter virus (Fig. 3A). In neither case did either HSP90 inhibitor reduce NLuc expression at 48 hours post-infection, indicating that there is no need for HSP90 in these early steps in the infection process. By contrast, geldanamycin potently inhibited replication of a subgenomic HAV RNA replicon (HAV-FLuc) (21) following its transfection into cells (Fig. 3B). This RNA replicon lacks sequence encoding the capsid proteins, and its replication does not involve viral assembly, cellular entry, or egress. These results thus point to HSP90 activity being required either for translation or replication of the HAV RNA genome. Previous studies show that although HSP90 activity is required for the proper folding and assembly of capsid proteins, geldanamycin does not inhibit replication of similar luciferase-expressing enterovirus or aphthovirus RNA replicons (5, 12). We confirmed that geldanamycin fails to inhibit luciferase expression from a subgenomic rhinovirus B14 replicon, even at very high concentrations (1 µM) (Fig. 3B). We also found that geldanamycin minimally inhibits replication of a subgenomic human parechovirus A1 replicon, representing a fourth genus within the *Picornaviridae* (Fig. 3B). Thus, picornaviruses vary in their requirement for HSP90 chaperone activity, with hepatoviruses being very different from other genera in requiring HSP90 for translation or replication of their RNA genomes.

Translation of the positive-sense HAV RNA genome is 5′ m^7^G cap-independent and initiated under the control of a structurally and functionally unique internal ribosome entry site (IRES) residing within the 5′ untranslated segment of the genome (22, 23). To determine whether HSP90 activity is required for the activity of this IRES, we transfected cells with a reporter plasmid that expresses transcripts containing the HAV IRES, a split green fluorescent protein (GFP) sequence, and intronic sequences driving the production of back-spliced circular RNA (circRNA) (22) (Fig. 3C). GFP can be synthesized from these transcripts only after the continuity of the GFP sequence has been restored by back-splicing, and since the back-spliced RNA has no free ends, translation is entirely dependent upon internal entry of ribosomes directed by the IRES. Notably, neither geldanamycin nor 17-AAG inhibited GFP synthesis from these circRNA transcripts, indicating that HAV translation is not dependent upon HSP90 activity (Fig. 3C). This suggests that the chaperone activity of HSP90 is required for a later step in the replicative cycle of the virus.

Following RNA translation and polyprotein synthesis, a crucial step in HAV replication involves the processing of the polyprotein by 3C^pro^, the only protease expressed by the virus. To determine whether HSP90 is required for 3C^pro^ protease function, we treated cells transfected with a vector expressing the 3ABCD segment of the polyprotein (Fig. 3D) with an amino-terminal HA tag (HA-3ABCD) with geldanamycin. 3ABCD is processed in *cis* by 3C^pro^ into a stable 3ABC intermediate (24) (Fig. 3D). At a concentration of 1 µM, geldanamycin modestly reduced the amount of 3ABC product without any visible accumulation of the 3ABCD substrate, which if misfolded may have been subject to proteasome-mediated degradation (Fig. 3E). However, there was no inhibition of 3ABCD processing by geldanamycin at a concentration of 0.1 µM (Fig. 3E), which is 30- to 40-fold above the IC_50_ of the compound for HAV replication (Fig. 1D and 3B). To evaluate *trans*-processing by 3C^pro^, we transfected cells with vectors expressing HA-3ABC and mitochondrial-antiviral signaling protein fused at its amino terminus to GFP (Fig. 3F). The 3ABC processing intermediate has protease activity and efficiently cleaves MAVS, effectively blocking MAVS-dependent antiviral signaling (25). Immunoblot analysis showed that geldanamycin had no impact on the cleavage of GFP-MAVS by 3ABC (Fig. 3F). Collectively, these data suggest that the chaperone activity of HSP90 is not required for the proper folding and proteolytic activity of 3C^pro^, either in *cis* or in *trans*. Consistent with this, immunoblots of lysates from infected cells treated with geldanamycin revealed concentration-related decreases in both structural (VP1) and nonstructural proteins (3ABC), with no detectable accumulation of precursor P1 or P3 (3ABCD) polypeptides (Fig. 3G).

### LFQ proteomic analysis of proteins interacting with HSP90

**Fig 4:**
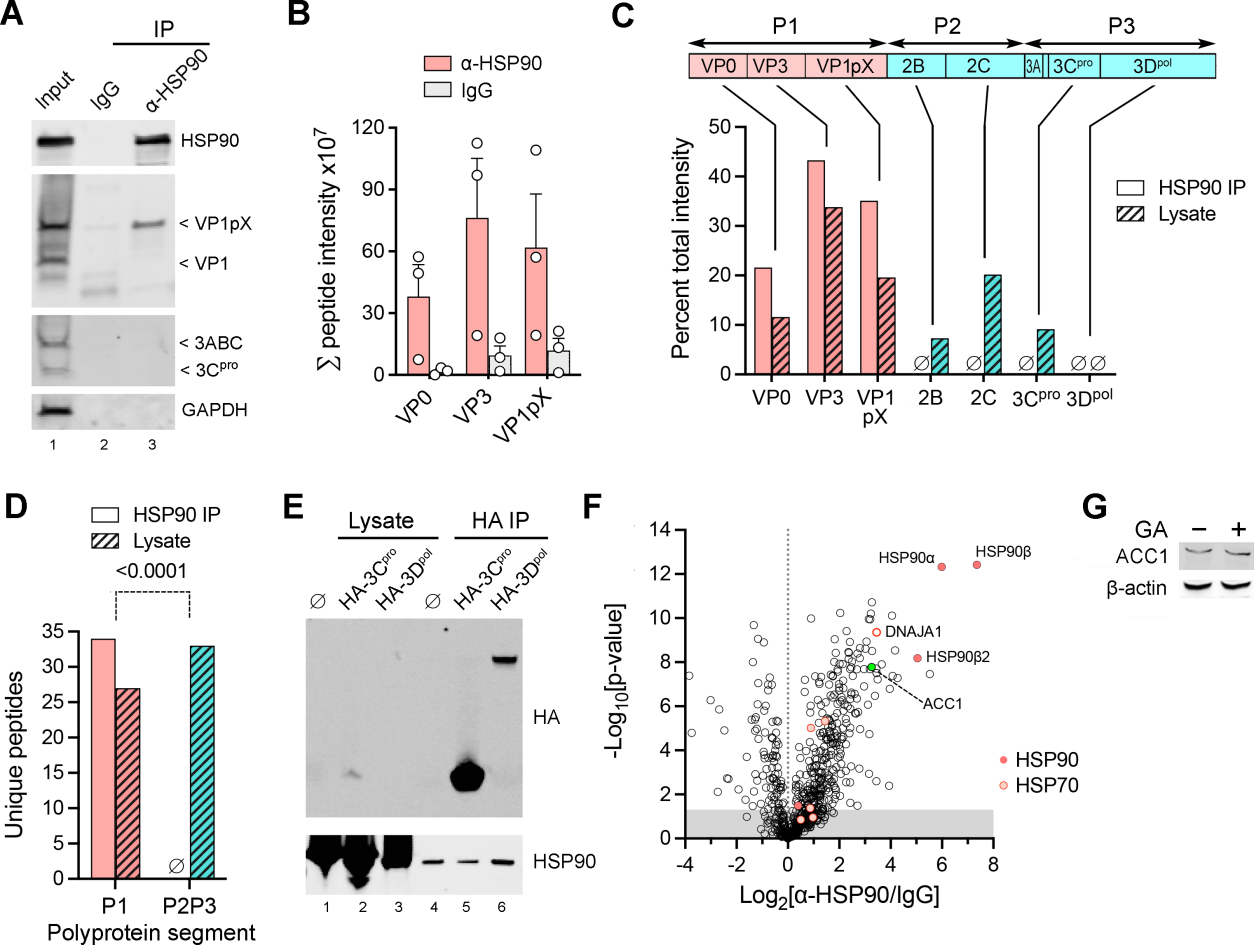
Label-free proteomics analysis of HSP90-interacting proteins. (A) Immunoblots of proteins immunoprecipitated (IP) from 18f-infected Huh-7.5 cell lysates with anti-HSP90 antibody (α-HSP90) or isotype control antibody (IgG). The lysate is in lane 1. (B) Summed MS intensities of peptides derived from VP0, VP2, and VP1pX in proteins pulled down from infected Huh-7.5 cell lysates with anti-HSP90 versus isotype control IgG antibody. Data are means ± SD, *n* = 3 independent precipitates; IP versus lysate *P* = 0.0037 by two-way ANOVA. (C) Distribution of HAV-related peptide intensities identified in anti-HSP90 pulldown products versus infected whole cell lysate. ∅, none identified. Peptide intensities were summed as in panel B, then computed as a percentage of the total. (D) Number of unique peptides derived from the structural (**P1**) versus nonstructural (P2P3) segments of the HAV polyprotein identified in the anti-HSP90 precipitate. *P*-value was calculated by Fisher’s exact test. (E) HSP90 immunoblot of anti-HA immunoprecipitates (HA IP) from cells ectopically expressing HA-3C^pro^ and HA-3D^pol^. ∅, mock transfection. (F) Volcano plot of 691 host cell proteins co-immunoprecipitating with HSP90 from HAV p16 virus-infected Huh-7.5 cells. The ratio of peptide MS intensities of proteins pulled down by anti-HSP90 versus isotype control IgG is plotted against *P*-value by *t*-test. ACC1, acetyl-CoA carboxylase 1. Shaded area, *P* > 0.05; *n* = 3. (G) Immunoblot of ACC1 in lysates of Huh-7.5 cells with or without 24 hours treatment with 200 nM geldanamycin (GA).

To gain an unbiased view of the interactions of HSP90 with both structural and nonstructural proteins expressed by HAV, we utilized label-free quantitative (LFQ) proteomics to characterize viral proteins that were co-immunoprecipitated from lysates of HAV-infected cells by antibody to HSP90 (Fig. 4A). Thirty-four unique HAV-derived peptides were identified by mass spectrometry in the anti-HSP90 immunoprecipitate (Table S2). These peptides were all derived from the major capsid proteins, VP2, VP3, and VP1pX, and were significantly more abundant in the anti-HSP90 versus IgG pulldown product (Fig. 4B). They included a peptide spanning the VP1-pX junction (polyprotein residues 753–769). Immunoblotting confirmed that VP1pX was present in the precipitate but not the fully processed VP1 protein found in naked nHAV particles (Fig. 4A). Importantly, no peptides derived from the nonstructural P2P3 segment of the polyprotein were identified in the anti-HSP90 pulldown products, despite the fact that peptides derived from 2B, 2C, and 3C^pro^ were abundant and readily detected by mass spectrometry in whole cell lysates of p16 virus-infected cells (Fig. 4C; Table S2). This difference in the distribution of unique peptides derived from the structural (P1) versus nonstructural (P2P3) segments of the polyprotein in the anti-HSP90 pulldown product versus the cell lysate was highly significant (*P* < 0.0001) (Fig. 4D). No 3D^pol^ polymerase-derived peptides were identified in either the anti-HSP90 pulldown or in the p16-infected cell lysate, reflecting the low abundance of the polymerase in infected cells (26). However, a co-immunoprecipitation experiment failed to demonstrate an interaction between HSP90 and either ectopically expressed 3D^pol^ or 3C^pro^ (Fig. 4E). These results suggest that HSP90 likely interacts with and thus may facilitate the folding of the HAV capsid proteins or their P1 polyprotein precursor, much as HSP90 promotes the folding of the poliovirus P1 polyprotein (5, 10). However, they leave unexplained why HSP90 activity is required for replication of the subgenomic viral RNA replicon, as shown above (Fig. 3B).

### Super-resolution immunofluorescence microscopy of HSP90

**Fig 5:**
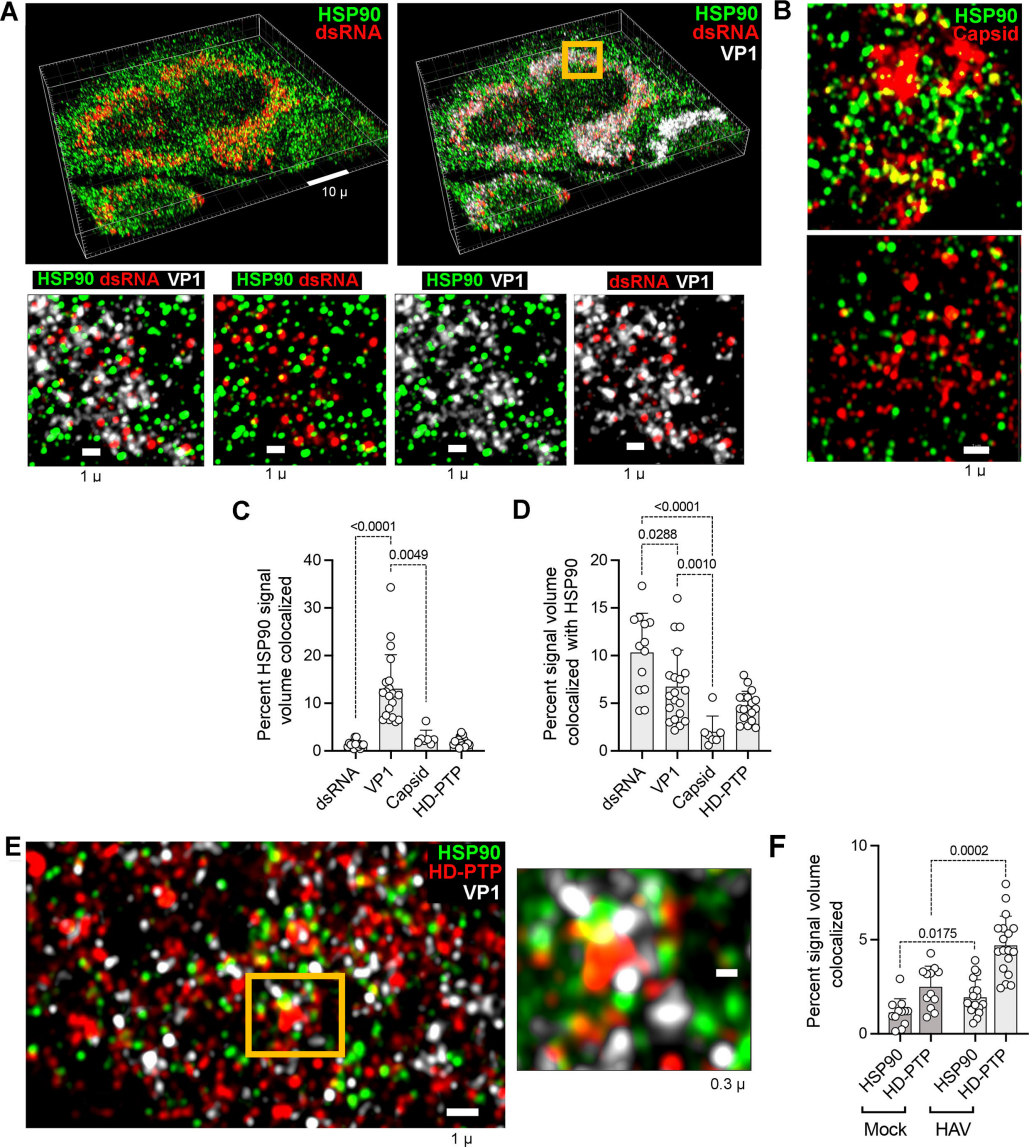
Airyscan super-resolution confocal immunofluorescence microscopy of Huh-7.5 cells infected with HM175/p16 virus for 7 days. (A) At the top are low magnification images of 3D volume reconstructions of cells labeled with antibodies to (left) HSP90 (green) and dsRNA (red, J2 antibody), or (right) HSP90 (green), dsRNA (red), and VP1 aa7-143 (white). Sections (0.55 µ) of the region highlighted in yellow are shown below labeled as indicated. (B) Sections from cells labeled with antibodies to HSP90 (green) and the assembled HAV capsid (K3-2F2, red). (C) Percent volume in 3D image reconstructions of infected cells containing HSP90 signal above an arbitrary threshold that also contains signal above thresholds for dsRNA, VP1 aa7-143, capsid (K3-2F2), and HD-PTP signals. (D) Percent volume containing dsRNA, VP1 aa7-143, capsid (K3-2F2), or HD-PTP signals above threshold that also contain HSP90 signal above threshold. (E) Section of a cell labeled with antibody to HSP90 (green), the ESCRT-associated protein HD-PTP (red), and VP1 (white). On the right is a detailed image of the region highlighted in yellow. (F) Percent shared volume colocalization of HSP90 with HD-PTP and vice versa in uninfected (mock) and p16 virus-infected (HAV) cells. *P*-values by unpaired two-sided *t*-test. Scale bars as labeled in each panel.

We used super-resolution Airyscan immunofluorescence microscopy to visualize the spatial relationship of HSP90 and double-stranded RNA (dsRNA), a reliable marker of viral replication organelles, VP1 protein labeled by an antibody raised to a recombinant VP1 fragment (amino acid residues 7–143), and HAV capsids labeled with a neutralizing anti-HAV monoclonal antibody that recognizes a conformational epitope, K3-2F2 (27, 28), in p16 virus-infected cells (Fig. 5A and B). The HSP90 signal was abundant and dispersed throughout the cytoplasm, whereas the dsRNA and VP1 signals generally clustered in regions near the nucleus consistent with replication organelles (Fig. 5A). Quantitative analysis of 3D reconstructions of these images revealed that only a small fraction of the volume occupied by HSP90 signal above threshold (mean 1.4% ± 0.8 SD) overlapped with the dsRNA signal, whereas a much greater proportion of the HSP90 signal (13% ± 7.2%) overlapped with VP1 signal (Fig. 5C). Conversely, 10% ± 4.0% of the volume occupied by the dsRNA signal and 6.7% ± 3.8% of the volume occupied by VP1 were also occupied by HSP90 signal (Fig. 5D). We also observed colocalization of HSP90 with capsid antigen labeled with K3-2F2 (Fig. 5C). This antibody is distinct from the VP1 antibody, as it recognizes only fully assembled HAV capsids (29). Importantly, the degree to which the capsid antibody signal colocalized with HSP90 (and vice versa) was significantly less than the colocalization of VP1 signal with HSP90: only 2.7% ± 1.6% of the HSP90 signal volume overlapped capsid antigen signal, and 2.0% ± 1.7% of capsid antigen signal overlapped HSP90 (Fig. 5C and D). Collectively, these results indicate that HSP90 is located in close proximity to replication organelles containing dsRNA and suggest a greater association of HSP90 with non-native VP1 protein than fully assembled capsids. The results are congruent with the label-free proteomics studies described above and provide further support for the notion that HSP90 acts as a chaperone in the folding and assembly of HAV capsid proteins.

Consistent with the presence of HSP90 in purified extracellular quasi-enveloped virions (19), we also found HSP90 colocalized with His domain-containing protein tyrosine phosphatase (HD-PTP), a cellular protein that plays a crucial role in the vesicle-mediated export of HAV capsids from infected cells (Fig. 5E). HD-PTP is an accessory component of the endosomal sorting complexes required for transport (ESCRT) (30, 31). It acts specifically at endosomal membranes where it facilitates the ESCRT-dependent sorting of cargo into multivesicular endosomes (MVE). HD-PTP interacts directly with the pX domain of VP1pX and is required for the loading of HAV capsids into intralumenal vesicles within MVE, a key step in the export of quasi-enveloped virus from infected cells (32, 33). Only 2.5% ± 1.1% of the volume occupied by the HD-PTP signal was also occupied by HSP90 in uninfected cells, but this increased to 4.7% ± 1.6% in infected cells (*P* = 0.0002) (Fig. 5F). Similarly, the reciprocal volume colocalization of HSP90 with HD-PTP increased from 1.1% ± 0.7% to 1.9% ± 0.9% (*P* < 0.0175) in HAV-infected cells (Fig. 5F). These results are consistent with some HSP90 remaining associated with the mature capsid during its HD-PTP-dependent loading into MVEs for export from the cell.

### Host cell proteins interacting with HSP90

In addition to the HAV proteins identified by mass spectrometry in the anti-HSP90 immunoprecipitates from infected cells (Fig. 4B through D), there were a total of 691 host cell proteins (Table S4). Of these, 88 were more than fourfold enriched in anti-HSP90 versus control IgG pull-down products (Fig. 4F). These included three distinct HSP90 proteins (HSP90β, HSP90β2, and HSP90α, with HSP90β most abundant) and the cochaperone DNAJA1. HSP70 proteins were also represented in the anti-HSP90 pull-down product, but at lesser levels (Table S4). Among the most enriched of the cellular proteins in the HSP90 pull-down products was acetyl-CoA carboxylase 1 (ACC1, *P* = 1.6 × 10^−8^), a known HAV host factor (18, 34) (Fig. 4F). Eighty-five unique peptides derived from ACC1 were identified in the anti-HSP90 precipitate, representing 46.1% coverage of the ACC1 amino acid sequence. By contrast, no ACC1 peptides were identified in whole cell lysates of infected cells by mass spectrometry. These data suggest that ACC1 likely exists in a complex with HSP90. This enzyme was identified as a host factor for HAV in the CRISPR screen, and it is required for host cell fatty acid synthesis, essential for HAV RNA replication (18, 34). Although 24 hours exposure to 200 nM geldanamycin did not reduce the abundance of ACC1 in Huh-7.5 cells (Fig. 4G), these data raise the possibility that ACC1 may be an HSP90 client. If so, HSP90 inhibitors might block the replication of HAV, not by interfering with the functional maturation of viral proteins, but rather by hindering HSP90-dependent acquisition of function by an essential host factor, ACC1.

## DISCUSSION

Previous studies have shown that many different viruses from widely diverse virus families are dependent upon the HSP90 chaperone network for efficient replication (3, 4). The specific client proteins that require HSP90 to acquire the functions necessary for viral replication are generally not well defined but are equally broad and varied, ranging from structural protein precursors and fully assembled capsids to polymerases. Here, we show, despite a previous claim to the contrary (16), that HAV is no exception. Like many other previously studied viruses, the replication of HAV is potently inhibited, both in cell culture (Fig. 1) and *in vivo* within the liver of *Ifnar1^-/-^* mice (Fig. 2), by chemical inhibitors targeting the ATP binding site of HSP90. The inhibitory IC_50_ concentrations of both geldanamycin and its chemical analog 17-AAG are approximately 30-fold lower than the cytotoxic CC_50_ concentrations of these drugs in hepatocyte-derived cells (Table 1), suggesting a high degree of specific dependence upon the chaperone network.

We confirmed that HAV is relatively resistant to geldanamycin in fetal rhesus kidney cells, as noted previously by Aragonès et al*.* (16) (Fig. 1E). The HAV genome possesses extreme codon bias, with codon usage similar to that of viruses infecting invertebrate hosts (35). This suboptimal codon usage may slow the translation of HAV RNA, leading to the suggestion that the slow synthesis of viral proteins could facilitate co-translational folding and thus explain the apparent lack of dependence on the HSP90 chaperone system in FRhK4 cells (10, 16). However, replication and nanoluciferase expression from the HAV reporter virus genome proceed with similar kinetics during the first 48 hours of infection in FRhK4 and Huh-7.5 cells, suggesting there is no great difference in the rate of HAV translation in these cells. Moreover, we found that geldanamycin is also much less active in inhibiting poliovirus replication in FRhK4 cells compared to Huh-7.5 cells (Fig. 1F). Geldanamycin is also less cytotoxic in FRhK4 than Huh-7.5 cells (Fig. 1E and Table 1), suggesting it may be taken up less efficiently into FRhK4 cells or that there are quantitative or qualitative differences in HSP90 machinery in these fetal cells. Whatever underlies the difference in geldanamycin sensitivity in these different cell types, our data dispel earlier suggestions that HAV is unique among picornaviruses in replicating independently of HSP90.

Despite our efforts to identify a key step in the HAV replication cycle that is blocked by inhibiting HSP90, how HSP90 contributes to the production of new virus remains unclear and is likely multifactorial. HSP90 activity is known to be required for the processing of the P1 capsid protein precursor polypeptides of enteroviruses and aphthoviruses (5, 12). When HSP90 is inhibited, the poliovirus P1 capsid protein precursor accumulates despite 3C^pro^ protease activity, indicating that chaperone-facilitated folding is required for P1 substrate competence (5). Similarly, HSP90 inhibitors block 2A processing of a recombinant FMDV P1-2A protein and the subsequent assembly of 14S pentamers, the first step in capsid assembly (10). Our data show that HSP90 is associated with the capsid proteins of HAV, both within infected cells (Fig. 4B through D) and, in previous studies, extracellular quasi-enveloped virions (19). This suggests that the folding and assembly of HAV capsid proteins may similarly require HSP90. However, unlike the previous studies with poliovirus and FMDV (5, 12), there was no accumulation of HAV P1 capsid protein precursor relative to VP1pX or VP1 when HSP90 was inhibited (Fig. 4G). This suggests that 3C^pro^ processing of the precursor may be preserved in the absence of HSP90 activity, but it does not exclude a requirement for HSP90 in subsequent steps in capsid assembly.

In contrast to the inhibition of enterovirus and aphthovirus replication by geldanamycin, inhibiting HSP90 has negligible effects on the replication of subgenomic enterovirus or aphthovirus RNA replicons (5, 12). Although recent single-cell analyses of poliovirus-infected cells suggest that HSP90 inhibitors may significantly slow the synthesis of viral RNA (36), these studies of replicon RNAs indicate that HSP90 activity is not required for efficient translation, replication organelle assembly, or synthesis of negative- and positive-strand enteroviral and aphthoviral RNAs. HAV is clearly different. We confirmed that geldanamycin does not inhibit subgenomic enterovirus (rhinovirus) RNA replication in Huh-7.5 cells and also found that a human parechovirus replicon was resistant to geldanamycin (Fig. 3B). By contrast, the geldanamycin IC_50_ for a similar HAV replicon was 29.1 nM, close to that for the virus itself (Fig. 3B and Table 1). This requirement for HSP90 in HAV genome replication may explain, at least in part, the lower IC_50_ of geldanamycin for inhibiting replication of HAV (8.7–11.8 nM in Huh-7.5 cells) compared with poliovirus (183 nM in Huh-7.5 cells and 110 nM in HeLa S3 cells [5]).

The HSP90 client required for HAV RNA replication in these experiments is uncertain. We found that the HAV 3C^pro^ protease functions independently of HSP90 (Fig. 3E through G), and our proteomics studies failed to identify any nonstructural protein interacting with HSP90 (Fig. 4C and D). Surprisingly, however, peptides derived from the RNA-dependent RNA polymerase 3D^pol^ were not identified by mass spectrometry in these experiments, either in whole lysates or in HSP90 immunoprecipitates (Fig. 4C). Previous studies show that HAV 3D^pol^ is expressed only at low abundance and suggest that it may be subject to ubiquitination and proteasome-mediated degradation (26). Multiple RNA and DNA polymerases and reverse transcriptases expressed by other viruses are known to be HSP90 clients (3). Thus, it remains possible that the very low abundance HAV 3D^pol^ polymerase may require chaperone-mediated folding for its activity, even though we found no evidence for an interaction between endogenous HSP90 and recombinant 3D^pol^ expressed ectopically in cells (Fig. 4E). It is noteworthy that previous efforts to express a functional, recombinant HAV 3D^pol^ were unsuccessful. In contrast to the poliovirus polymerase, the HAV 3D^pol^ product of 3ABCD (P3) autocleavage lacked polymerase activity and was virtually completely insoluble (37).

An alternative possibility is that HSP90 chaperone activity may be required for the functional maturation of an essential HAV host factor. The 88 host cell proteins we found to be highly enriched in HSP90 immunoprecipitates from infected cells (Table S4) included 4 of the top 41 high-probability HAV host factor candidates identified in our previous genome-wide CRISPR screen: DNAJA1, the translation initiation factors eIF3F and eIF3M, and ACC1 (18). ACC1 catalyzes the initial, rate-limiting step in *de novo* fatty acid synthesis and is essential for efficient HAV RNA replication (34, 38). A chemical inhibitor of ACC1 (firsocostat) potently inhibits HAV replication in cell culture (34). ACC1 is a very large protein with 2,346 amino acid residues (266 kDa). We cannot exclude the possibility that inhibiting HSP90 negatively impacts its catalytic activity, despite the apparent absence of ACC1 degradation in geldanamycin-treated cells (Fig. 4G). Geldanamycin and 17-AAG had no effect on cap-independent translation directed by the HAV IRES in circular RNA reporter assays (Fig. 3C), suggesting that the inhibition of replicon amplification does not result from misfolding of eIF3F or eIF3M.

In conclusion, our data show that HSP90 is essential for efficient HAV replication despite a prior report to the contrary (16). HSP90 interacts with HAV capsid proteins and seems likely to be required for the assembly of the capsid and its interaction with the ESCRT adapter, HD-PTP, an essential step in cellular egress of the virus (32). HSP90 is also required for replication of the HAV RNA genome independent of any interaction with its capsid proteins. Although not approved for any viral infection, HSP90 inhibitors have long been touted as potential broad-spectrum antiviral agents, and like poliovirus and EV-A71 (5, 13), HSP90 inhibitors have significant antiviral activity against HAV in a murine model of infection.

## MATERIALS AND METHODS

### Cells

HAV was propagated in human hepatoma-derived Huh-7.5 or Huh-7.5.1 cells (39, 40) maintained in DMEM with 3%–10% fetal bovine serum and 1% penicillin/streptomycin. These cells tested negative for mycoplasma by PCR assay (LookOut Mycoplasma PCR Detection Kit, Sigma). PH5CH8 cells with double genetic knockout of MAVS and IRF1 (17) were provided by Daisuke Yamane, Tokyo Metropolitan Institute of Medical Sciences. 293T, FRhK4, and A549 cells were obtained from the American Type Culture Collection.

### Virus, RNA replicons, and plasmids

The cell-culture-adapted HM175 strain HAV variants p16 (GenBank KP879217.1) (41) and 18f (KP879216.1) (42), subgenomic HAV-FLuc replicon RNA constructed from the 18f virus genome (21), 18f-NLuc reporter virus (17), PV1-NLuc reporter virus (43), and human parechovirus A1 replicon (PeV-1A-NLuc) (44) have been described previously. Murine infections were carried out with the mouse-passaged HM175 strain of HAV (20). Plasmid vectors expressing HA-tagged HAV proteins derived from HM-175/18f virus, including catalytically active and inactive 3ABC-C172A (25), GFP-MAVS (45), and the circRNA HAV IRES reporter plasmid (22), have been previously described.

### Chemicals

Geldanamycin was purchased from MedChem Express (HY-15230) or Seleck Chemicals (S1141), and 17-AAG from MedChem Express (HY-10211) or Selleck Chemicals (S1141). JG98 (HY-117282), VER155008 (HY-10941), and 116-9e (HY-116683) were purchased from MedChem Express, and HSP990 (S7097), STA9090 (S1159), and TAS116 (S7716) from Selleck Chemicals. The Anti-Virus Compound Library (L1700) was purchased from TargetMol, Wellesley Hills, USA.

### Antibodies

The following antibodies were used in these studies: anti-HSP90 (Santa Cruz sc-13119 or Proteintech 13171-1-AP), anti-dsRNA J2 clone (Exalpha, 10010500), anti-HA (Fortis Life, A190-138A), anti-GFP (Santa Cruz, sc-9996), anti-ACC1 (Proteintech, 21923-1-AP), anti-GAPDH (Proteintech, 10494-1-AP): anti-HAV VP1 (LSBio, LS-C137674), anti-HAV 3C (gift from Prof. Verena Gauss-Müller, University of Lübeck), anti-β-actin (Sigma, A2228), and anti-IBA1 (Wako Pure Chemical Industries, 019-19741).

### RT-qPCR quantitation of HAV RNA

HAV RNA was extracted from liver tissue using TRIzol reagent (Invitrogen Life Technologies) following the manufacturer’s protocol and from cultured cells using the RNeasy Kit (Qiagen). HAV RNA was extracted from serum or fecal samples using the QIAamp Viral RNA Isolation Kit (Qiagen). RT-qPCR was carried out with the THUNDERBIRD Probe One-step qRT-PCR kit (Toyobo) and StepOnePlus Real-Time PCR System (Thermo Fisher Scientific). The forward primer was 5′-AGGGTAACAGCGGCGGATAT-3′, and the reverse primer was 5′-ACAGCCCTGACARTCAATYCMCT-3′. The TaqMan probe was FAM-5′-AGACAAAAACCATTCAACRCCGRAGGAC-3′-TAM. The one-step RT-qPCR running protocol included 5 min at 50°C, 30 s at 95°C, 3 s at 95°C, and 30 s at 60°C for 40 cycles.

### Luciferase assays

Cells were lysed in 1× passive lysis buffer (Promega, #E1941) for 15 min at room temperature, then lysates were transferred to opaque white 96-well plates (Corning, #3912). NLuc activity was quantified using the NLuc GLOW Assay kit (Nanolight Technology, #325), and FLuc assays were carried out with the Luciferase Assay System (Promega, E1500). Luminescence was measured using a Biotek Synergy II multi-mode plate reader (BioTek Instruments).

### Transfections

Viral and replicon RNAs were transcribed *in vitro* from plasmid DNA as described previously (46). RNA transfections were carried out with the Trans-IT mRNA reagent (Mirus Bio, #MIR 2225) and plasmid transfections with Lipofectamine 3000 Transfection Reagent (Invitrogen, #L3000008) following the manufacturers’ suggested protocols.

### Chemical screen for antiviral compounds with activity against HAV

Huh-7.5.1 cells were inoculated with the 18f-NLuc reporter virus at a multiplicity of 100 GE (genome equivalents) per cell. The cells were washed with medium 6 hours later, then refed with medium containing 10 µM of each compound in the TargetMol Anti-Virus Compound Library (TargetMol, L1700) or vehicle only (1% DMSO). Cells were assayed for viability using the Cell TiterGlo Luminescent Cell Viability Assay (Promega, G7570) 72 hours after the addition of compound, and supernatant fluids were collected for inoculation onto naive Huh-7.5.1 cells for a second cycle infection. Six hours following inoculation with the supernatant fluids, these newly infected cells were washed with medium, then cultured in the absence of any compound. Nanoluciferase activity was measured 48 hours later. Vehicle (DMSO) was used as the negative control and interferon-γ (Kyowa Pharmaceutical Industry Ltd., 1 U/mL) as a positive control.

### Antiviral activity of 17-AAG in *Ifnar1^-/-^* mice

Mice were bred and housed at the National Institute of Infectious Diseases, Japan, in accordance with the policies and guidelines of the Institutional Animal Care and Use Committee (IACUC). *Ifnar1^-/-^* mice were provided by Dr. S. Morikawa (47). The inoculum was generated from the liver of an *Ifnar1^-/-^* mouse infected with 12th murine passage HM175 virus (20) by homogenizing the tissue in PBS, followed by centrifugation at 10,000 × *g* for 30 min as described previously (48). *Ifnar1^-/-^* mice were infected at 8–12 weeks of age by intravenous inoculation of 10^6^ GE of virus and subsequently administered 17-AAG (1 mg/mouse/day) or vehicle control (DMSO) by intraperitoneal injection. Feces, serum, and tissues were collected on day 7 following infection. Liver tissue was fixed in 10% neutral phosphate-buffered formalin for 24 hours, then stored in 70% ethanol until processed for histology. Serum alanine aminotransferase activities were measured using the DRI-CHEM SLIDE NX700 analyzer and GPT/ALT-P III (Fujifilm). Fixed tissues were embedded in paraffin, sectioned, and stained with hematoxylin and eosin. For immunohistochemical staining of macrophages, sections were processed at 95°C for 20 min in Bond Epitope Retrieval Solution (pH 6; H1, AR9661, Leica Biosystems) in an automated stainer (Leica Bond RXm; Leica Biosystems, Germany), then labeled with anti-IBA1 antibody (Wako Pure Chemical Industries, 019-19741) using the BOND Polymer Refine Detection (DS9800, Leica Biosystems) system and Nichirei-Histofine Simple Stain Mouse MAX PO (Nichirei Biosciences) for secondary detection. Nuclei were counterstained with hematoxylin. IBA1-positive cells were enumerated using inForm Tissue Analysis Software (AKOYA Biosciences) following whole-slide imaging (PhenoImager Fusion, Akoya Biosciences).

### RNA replicon assays

Freshly transcribed RNA (0.3 µg) was transfected into Huh-7.5 (HAV-FLuc and RV-B14-FLuc) or A549 cells (PeV-A1) in 24-well plates using the TransIT-mRNA kit (Mirus Bio) and treated with the indicated amount of geldanamycin. Luciferase activities were measured 24 hours post-transfection.

### HAV circRNA IRES reporter assay

Cells were transfected with the circRNA HAV IRES reporter plasmid (22), which contains a split GFP sequence and intronic sequences that drive the production of back-spliced circRNA containing the HAV IRES upstream of the reconstituted GFP sequence (49–51). Cap-independent HAV IRES-directed translation was monitored by immunoblot analysis of GFP expression.

### Label-free quantitative proteomics of HSP90-associated and cytosolic proteomes of p16 virus-infected cells

Triplicate cultures of Huh-7.5 cells were infected with p16 virus for 7 days, then harvested in lysis buffer (150 mM KCl, 25 mM Tris-HCl, pH 7.4, 5 mM EDTA, 1% Triton X-100, 5 mM DTT, and Complete Protease Inhibitor Cocktail [Roche]). Lysates were sonicated and centrifuged, and supernatants were incubated with anti-HSP90 antibody (Santa Cruz, sc-13119 AC) or isotype control IgG (Santa Cruz, sc-2343) conjugated agarose beads at 4°C for 2 hours. Beads were washed four times in lysis buffer, and proteins eluted in SDS-PAGE sample buffer were subjected to in-gel digest. To characterize the cytosolic proteome, the cytosol of infected cells was purified (CelLytic NuCLEAR Extraction Kit, Sigma), then the proteins were solubilized, acetone-precipitated, and resolubilized in 8 M urea. Proteins precipitated with anti-HSP90 or cytosol proteins were subjected to tryptic digestion, then the peptides were extracted and desalted on home-made C18 stage-tips. The clean peptides were dissolved in 0.1% formic acid and analyzed on a Q-Exactive HF-X mass spectrometer coupled with an Easy nanoLC 1200 System (ThermoFisher Scientific, San Jose, CA, USA). Peptides were loaded onto a nanoEase MZ HSS T3 Column (100 Å, 1.8 µm, 75 µm × 250 mm, Waters). Analytical separation of the anti-HSP90-pulldown digests was achieved with a linear gradient of 5%–30% buffer B over 29 min, and 30%–45% buffer B over 6 min at a 250 nL/min flow rate, followed by a ramp to 100% B in 1 min and 14 min wash with 100% B, where buffer A was aqueous 0.1% formic acid, and buffer B was 80% acetonitrile and 0.1% formic acid. For the cytosolic proteome, peptide separation was achieved with a linear gradient of 5%–30% buffer B over 100 min, and 30%–45% buffer B over 20 min at a 250 nL/min flow rate, followed by a ramp to 100% B in 1 min and a 14 min wash with 100% B.

LC-MS experiments were performed in a data-dependent mode with full MS and with a resolution of 60,000 at *m/z* 200, followed by high-energy collision-activated dissociation-MS/MS of the top 15 most intense ions with a resolution of 15,000 at *m/z* 200. High-energy collision-activated dissociation-MS/MS was used to dissociate peptides at a normalized collision energy of 27 eV in the presence of nitrogen bath gas atoms. There were three technical LC-MS replicates for each anti-HSP90 precipitated sample and two technical replicates for each cytosolic sample.

Mass spectra were processed, and peptide identification was carried out using MaxQuant software version 1.6.10.43 (Max Planck Institute, Germany). Protein database searches were performed against the UniProt human protein sequence database (UP000005640) and p16 virus proteins (P08617). A FDR for both peptide-spectrum match and protein assignment was set at 1%. Search parameters included up to two missed cleavages at Lys/Arg on the sequence, oxidation of methionine, and protein N-terminal acetylation as a dynamic modification. Carbamidomethylation of cysteine residues was considered a static modification. Peptide identifications were reported by filtering reverse and contaminant entries and assigning them to their leading razor protein. LFQ was carried out with MaxQuant. Data processing and statistical analysis were done with Perseus version 1.6.10.50. Protein quantitation was carried out on technical replicates, using two-sample *t*-test statistics with a *P*-value of 0.05 considered a statistically significant fold difference in protein abundance for anti-HSP90 versus isotype control immunoprecipitates.

### Super-resolution Airyscan immunofluorescence microscopy

Huh-7.5 cells infected with p16 virus for 7 days were visualized in 35 mm glass bottom dishes with 14 mm bottom wells (Cellvis, Cat# D35-14-1.5-N). Cells were fixed with 4% paraformaldehyde for 12 min, washed twice with PBS, and incubated with a blocking/permeabilization solution containing 5% goat serum and 0.1% saponin in PBS for 1 hour at room temperature. Dishes were incubated for 1 hour at room temperature with primary antibody diluted in PBS with 1% BSA and 0.1% saponin, then washed with PBS containing 0.05% saponin, followed by incubation with appropriate secondary antibodies for 1 hour. Nuclei were counterstained with Hoechst 33342 (Invitrogen) for 10 min at room temperature, and the dishes were then washed three times with PBS. Imaging data were recorded in super-resolution mode on a laser-scanning confocal Zeiss 880 microscope (Carl Zeiss AG, Oberkochen, Germany) equipped with an Airyscan detector and controlled by Zen Blue 3.0 software. The microscope was mounted on a Zeiss Inverted Axio Observer Z1 base equipped with a Definite Focus unit to ensure focus plane stability. A Plan‐Apochromat 63×/1.40 Oil DIC M27 objective (Zeiss) was used for all imaging. For each image plane, fluorescence signals were recorded sequentially at different wavelengths in super-resolution mode using appropriate laser excitation and filter sets: for DAPI/ Hoechst, excitation: 405 nm, emission: filter BP 420-480 + BP 495-550; for Alexa488, excitation: 488 nm, emission filter BP 420-480 + BP 495-550; for Alexa568/594, excitation: 561 nm, emission filter BP 420-480 + BP 495-620; and for Alexa647, excitation: 633 nm, emission filter BP 570-620 + LP 645. Fields of view were selected randomly, maintaining identical imaging conditions for samples in each experiment. The number of X-Y pixels on the frame was set to “optimal” in the Zeiss software controller. The distance between images in the Z direction during Z-stack recording was 0.180 µm. Immediately after recording, the raw data were processed by Zen Blue software to produce a super-resolution image with 16-bit depth.

### Quantitative image analysis

Imaris software (version 10, Oxford Instruments) was used for 3D visualization of recorded Airyscan images and to analyze colocalization of fluorescent signals. Following background subtraction, the intensity thresholds of fluorescence signals were selected for each fluorescence channel separately using a semi-automatic procedure with the condition that the threshold should not be less than 5% of maximum channel intensity. The Imaris colocalization module was used to identify all voxels above the threshold for each fluorescence channel separately and the percentage of voxels for each fluorescence channel that were colocalized with fluorescence signals from another channel. New channels containing colocalization data were created for each pair of fluorescent signals (e.g., HSP90 with dsRNA or HD-PTP or capsid) and subjected to statistical analysis.

### Statistical analysis

Unless otherwise stated, statistical significance was assessed by unpaired, two-sided *t*-test or ANOVA. All statistical calculations were carried out using Prism 10.4.1 software (GraphPad).

## Data Availability

The data described in the article are available in the figures or supplemental tables.

## SUPPLEMENTAL MATERIAL

Tables S1 to S4 jvi.00502-25-s0001.xlsx Table S1. 18f-NLuc screen for chemical inhibitors of HAV replication. Table S2. HAV protein-related peptides identified in anti-HSP90 precipitates. Table S3. HAV protein-related peptides identified in p16 virus-infected cell lysate. Table S4. Host cell proteins co-immunoprecipitating with HSP90 in p16 virus-infected cells.

ASM does not own the copyrights to Supplemental Material that may be linked to, or accessed through, an article. The authors have granted ASM a non-exclusive, world-wide license to publish the Supplemental Material files. Please contact the corresponding author directly for reuse.
